# The specific host plant DNA detection suggests a potential migration of *Apolygus lucorum* from cotton to mungbean fields

**DOI:** 10.1371/journal.pone.0177789

**Published:** 2017-06-06

**Authors:** Qian Wang, Wei-Fang Bao, Fan Yang, Bin Xu, Yi-Zhong Yang

**Affiliations:** 1 College of Horticulture and Plant Protection, Yangzhou University, Yangzhou, China; 2 State Key Laboratory for Biology of Plant Diseases and Insect Pests, Institute of Plant Protection, Chinese Academy of Agricultural Sciences, Beijing, China; USDA Agricultural Research Service, UNITED STATES

## Abstract

The polyphagous mirid bug *Apolygus lucorum* (Heteroptera: Miridae) has more than 200 species of host plants and is an insect pest of important agricultural crops, including cotton (*Gossypium hirsutum*) and mungbean (*Vigna radiata*). Previous field trials have shown that *A*. *lucorum* adults prefer mungbean to cotton plants, indicating the considerable potential of mungbean as a trap crop in cotton fields. However, direct evidence supporting the migration of *A*. *lucorum* adults from cotton to mungbean is lacking. We developed a DNA-based polymerase chain reaction (PCR) approach to reveal the movement of *A*. *lucorum* between neighboring mungbean and cotton fields. Two pairs of PCR primers specific to cotton or mungbean were designed to target the *trn*L-*trn*F region of chloroplast DNA. Significant differences in the detectability half-life (DS_50_) were observed between these two host plants, and the mean for cotton (8.26 h) was approximately two times longer than that of mungbean (4.38 h), requiring weighted mean calculations to compare the detectability of plant DNA in the guts of field-collected bugs. In field trials, cotton DNA was detected in the guts of the adult *A*. *lucorum* individuals collected in mungbean plots, and the cotton DNA detection rate decreased successively from 5 to 15 m away from the mungbean-cotton midline. In addition to the specific detection of cotton- and mungbean-fed bugs, both cotton and mungbean DNA were simultaneously detected within the guts of single individuals caught from mungbean fields. This study successfully established a tool for molecular gut-content analyses and clearly demonstrated the movement of *A*. *lucorum* adults from cotton to neighboring mungbean fields, providing new insights into understanding the feeding characteristics and landscape-level ecology of *A*. *lucorum* under natural conditions.

## Introduction

Transgenic *Bacillus thuringiensis* (Bt) cotton was widely adopted for management of *Helicoverpa* species infestation in northern China during the late 1990s. As a result of reduced insecticide applications, the mirid bug *Apolygus lucorum* (Hemiptera: Miridae) has become the most dominant insect pest in the Yangtze River and Yellow River cotton-growing regions of China [1, 2]. As a polyphagous herbivorous pest, *A*. *lucorum* has more than 200 recorded host plant species and causes economic injury to cotton and other crops, including fruit trees and tea plants [3–5]. Such a broad host range together with its remarkable reproduction ability, long-distance dispersal capacity [6] and relatively inefficient control by predators [7] increases the destruction by these pests. To date, the suppression of mirid bug populations in the field still relies heavily on chemical pesticide applications, leading to problems associated with resistance development, pest resurgence and environmental pollution; thus, an environmentally friendly pest management strategy is required, such as “push-pull” habitat management that utilizes repellent plants and attractant trapping crops to manipulate the population density of pests on the target crop [8].

Large-scale field trials have shown that *A*. *lucorum* population abundance significantly differs among distinct habitats and host plants [9]. *Apolygus lucorum* adults typically exhibit clear preferences for particular plant species or plant growth stages [5]. From April to mid-June, *A*. *lucorum* reportedly prefers to settle and oviposit on *Medicago sativa* L and *Vicia faba* L. [4]. During the summer, the population density of *A*. *lucorum* on mungbean (*Vigna radiata* L.) appears to be significantly higher than that on cotton and other crops [10]. Combining the results from both field cages and open-field plots, Geng et al. (2012) reported that adult preference and nymph development are greater on mungbean than on cotton [11]. Additionally, the densities of these insects are significantly lower on cotton plots with mungbean strips than on cotton plots without mungbean [10]. These results suggest that mungbean cultivated near cotton fields might attract *A*. *lucorum* adults. Two-choice bioassay studies using a Y-shaped olfactometer demonstrate that *A*. *lucorum* adults prefer mungbean to cotton [6, 12]. However, this conclusion is based on laboratory observation and might not necessarily reflect reality under natural conditions. Direct evidence supporting the migration of *A*. *lucorum* from cotton to mungbean in open fields is lacking. In addition, studies identifying the host plants and determining dietary choices of this mirid bug rely heavily on conventional methods such as visual inspection. Because of the inconspicuous nature of symptoms after *A*. *lucorum* feeding, visual inspection for maceration symptoms to estimate feeding status might lead to false positive results in field surveys. Hence, a more accurate approach to assessing diet and to obtain direct information on the landscape-level movement of *A*. *lucorum* should be developed.

Diverse approaches have been developed to assess movement or identify arthropod host species [13–16]. Nevertheless, all of these useful methods appear to have inherent limitations. Molecular gut content analysis can overcome conventional methodological hurdles and provides an appealing alternative to address these issues [17, 18]. DNA-based gut content analyses are commonly used to track the trophic interactions between predators and prey [19–22]. Because multiple candidate DNA barcodes, including the internal transcribed spacer regions (ITS) of nuclear ribosomal DNA [23–25], the ribulose bisphosphate carboxylase gene large subunit (*rbc*L) [26–29], and the regions between tRNA for leucine (*trn*L) and tRNA for phenylalanine (*trn*F) (*trn*L-F regions) [30–35] on chloroplast DNA, have been successfully used to differentiate plant taxa, different DNA-based approaches have been developed to analyze the gut contents of herbivorous insects [18]. Diagnostic PCR employing general and specific primers [24, 25, 36–38] and the continual improvement of next generation sequencing (NGS) [39] allows the identity of phytophagous insect host plants to be recognized at the family [33, 40], genus [38, 41] and even species levels [23, 24, 42,43].

In this study, we designed specific primers targeting the *trn*L-*trn*F region and established two PCR assays for detecting the DNA of the two host plant species *G*. *hirsutum* and *V*. *radiata*. The specificity and sensitivity of both PCR assays were assessed. Subsequently, this newly developed plant DNA detection approach was employed to assess potential *A*. *lucorum* adult movement from cotton to mungbean fields under natural conditions.

## Results

### Assessment of the specificity of the designed plant primers

Plant-specific primers for *G*. *hirsutum* and *V*. *radiata* (Table 1) were designed based on an alignment of the *trn*L-*trn*F region among different host plant species of *A*. *lucorum*. To confirm their species specificity, the primers were tested against 31 non-target plants (Table 2). The results showed that all extracted plant DNA samples, except for the negative controls, exhibited a similar 120 bp band when general plant primers targeting the *trn*L region were used (S1 Fig), indicating the successful plant DNA extraction. The results of a cross-reactivity test revealed that the primers designed for cotton and mungbean amplified bands of 236 bp and 199 bp, respectively, and sequencing verification confirmed that these products were indeed cotton and mungbean. In addition, fragments at the same position were not observed when other plant DNA extracts were used (S2 Fig). These combined results indicate that the designed primers are species specific.

**Table 1:** Specific primer sequences (5'-3') target the *trn*L-*trn*F region of chloroplast DNA of cotton and mungbean.

Plant species	Forward primer sequence	Reverse primer sequence	Amplicon sizes (bp)
Cotton	GTTGAAGAAAGAATCGAATAGAATAG	ATAGACAGCAAACGGGCTTT	236
Mungbean	ATGTCAATACCGACAACAATGAA	AAATCCAAATTCCAATTTAGTTG	199

**Table 2:** Host plant species of *A*. *lucorum* used to develop the PCR-based plant DNA detection.

Family	Plant species
Amaranthaceae	*Amaranthus retroflexus* L
Asteraceae	*Artemisia scoparia* Waldst. et Kit.
	*Artemisia argyi*Lévl. et Vant.
	*Artemisia lavandulaefolia* DC.
	*Cirsium setosum* (Willd.) MB.
	*Helianthus annuus* L.
Balsaminaceae	*Impatiens balsamina* L.
Capparaceae	*Cleome gynandra* L.
Chenopodiaceae	*Salsola collina* Pall.
	*Chenopodium album* L.
Convolvulaceae	*Ipomoea purpurea* (L.) Roth
Euphorbiaceae	*Acalypha australis* L.
	*Ricinus communis* L.
Fabaceae	*Vigna radiata* (L.) Wilczek
	*Phaseolus vulgaris* L.
	*Astragalus adsurgens* Pall.
	*Medicago sativa* L.
	*Melilotus suaveolens* Ledeb.
	*Sophora japonica* Linn. var. japonica f. pendula Hort.
Moraceae	*Cannabis sativa* L.
	*Humulus scandens* (Lour.) Merr.
Malvaceae	*Gossypium hirsutum* L.
Poaceae	*Digitaria sanguinalis* (L.) Scop.
	*Echinochloa crusgalli* (L.) Beauv.
	*Setaria viridis* (L.) Beauv.
	*Zea mays* L.
Polygonaceae	*Fagopyrum esculentum* Moench
Rhamnaceae	*Ziziphus jujuba* Mill.
Rosaceae	*Malus pumila*
	*Prunus persica* L.
	*Pyrus bretschneideri* Rehd.
Solanaceae	*Solanum nigrum* L.
Vitaceae	*Vitis vinifera* L.

### Analysis of the plant DNA detectability half-life

**Fig 1:**
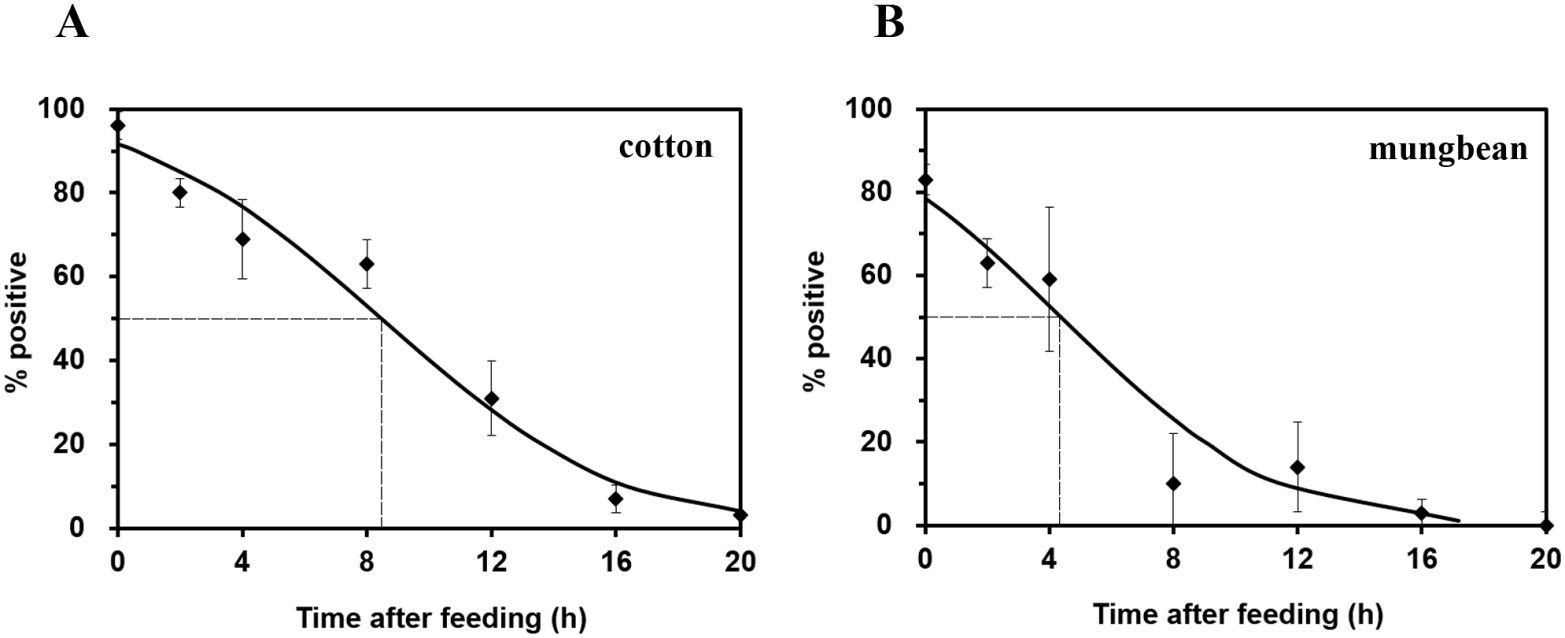
Detection of cotton (A) and mungbean (B) DNA in the guts of *A*. *lucorum* adults at different times after ingestion. Error bars at each point on the curves represent the standard error of three replicates. The model for the relationship between ingestion time (x) and % positive (y) was y = 100%×e^(2.27–0.26x)^/(1+e^(2.27–0.26x)^), F = 251.18, df = 2,5, *P*<0.0001, (A) and y = 100%×e^(1.53–0.37x)^/(1+e^(1.53–0.37x)^), F = 139.22, df = 2,5, *P*<0.0001 (B).

The PCR assay did not show clear amplification using general plant primers targeting *trn*L, suggesting that no host plant DNA remained in the gut and that adult *A*. *lucorum* were hungry enough to be readily fed. As shown in Fig 1, PCR amplicons were most consistently observed from adult *A*. *lucorum* immediately removed from plants, and declined with increasing digestion time. The maximum-life for plant detection did not reach 100% at time = 0 h and the value was significantly longer for cotton DNA than that for mungbean DNA. A negative exponential equation was constructed based on the detection curve, from which the detectability half-life (DS_50_) was calculated as 8.26 h for cotton, which was approximately two times longer than that of mungbean (4.38 h) (t = 4.53, df = 4, *P* = 0.01) (Fig 1A and 1B).

### Plant DNA detection rates in field-collected bugs and distance effects

**Fig 2:**
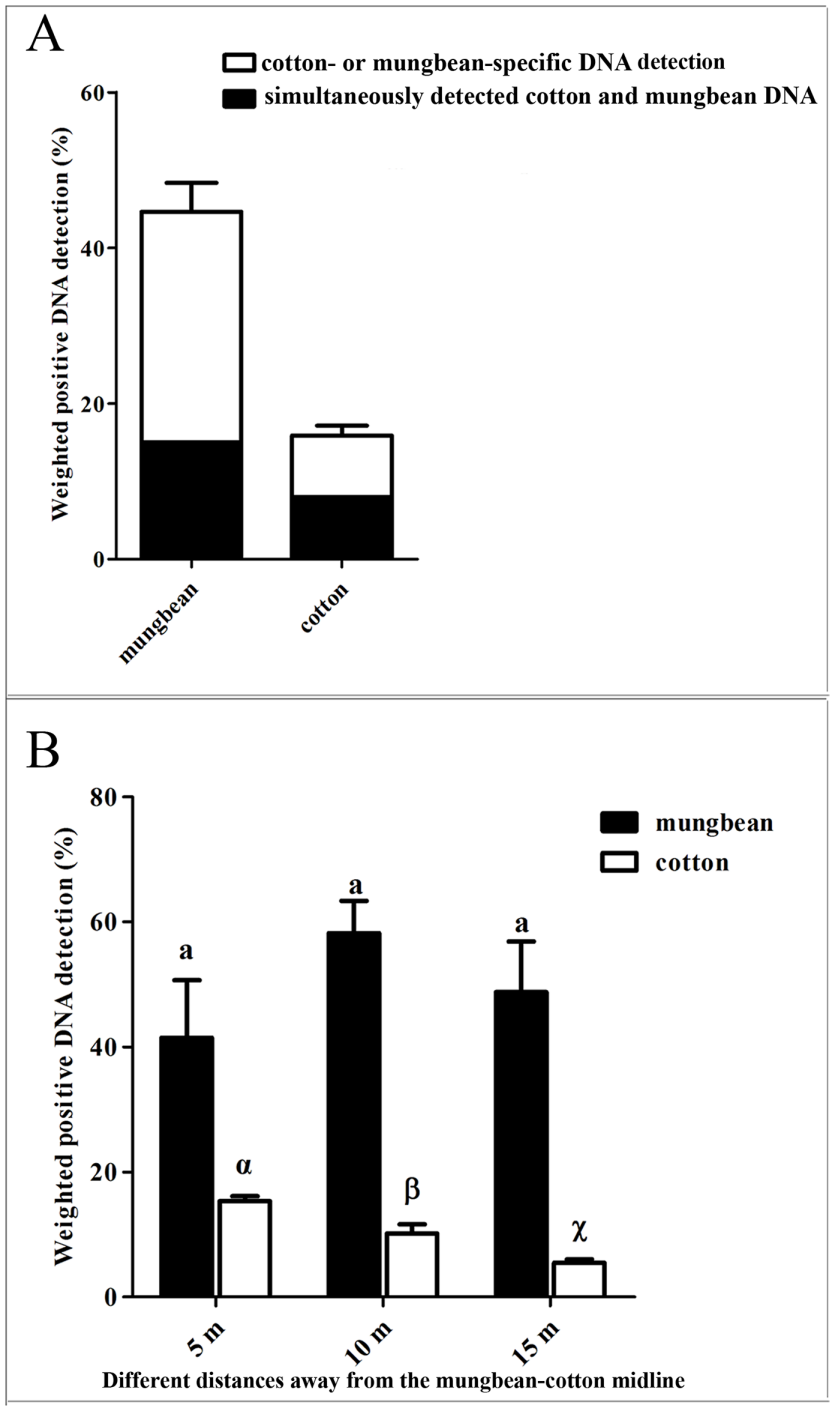
Detection rates of host plant DNA in mungbean field-collected bugs and the effect of distance. (A) The weighted DNA detection rates for mungbean and cotton in *A*. *lucorum* adult guts collected from mungbean fields were 44.64% and 15.65%, respectively. The black part of each column represents the percentage of simultaneously detected cotton and mungbean DNA. (B) Effect of different distances on the weighted DNA detection rates of mungbean and cotton in adult *A*. *lucorum* collected from mungbean plots. Error bars represent the standard error, and different letters (a) and (α, ß and χ) indicate no difference or a significant difference (*p*<0.05), respectively, among the tested samples.

To assess whether adult *A*. *lucorum* feed on mungbean and/or cotton in open fields and whether adult bugs in cotton fields migrate to mungbean plots, we collected adult *A*. *lucorum* from mungbean fields and performed molecular gut-content analyses using our newly developed species-specific DNA detection system. The average number of adult bugs specifically fed mungbean was estimated at 17.75, while the average number was estimated at 8.75 for those specifically fed cotton and 9.0 for those simultaneously feeding on both plants (n = 60 for each repetition). In total, the weighted detection rate was 44.64% for mungbean and 15.65% for cotton (Fig 2A). The simultaneous detection rate for both of these plants shown in the black part of each column in Fig 2A accounted for 50.70 percent of all identified cotton, which was much higher than the proportion (33.64%) of that in all the detection rates of mungbean (Fig 2A).

To evaluate the effect of distance on plant DNA detection rates, adult *A*. *lucorum* in mungbean plots 5, 10 and 15 m away from the mungbean-cotton midline were sampled and assessed. The results of this analysis revealed that the weighted detection rate of mungbean DNA in adult *A*. *lucorum* guts collected in mungbean plots was not significantly different for the three distances (F_2, 9_ = 1.24, *P =* 0.3332). However, the weighted detection rate of cotton DNA in adult *A*. *lucorum* guts varied for the three tested distances (F_2, 9 =_ 26.45, *P =* 0.0002), with the highest detection rates observed at 5 m, followed by 10 m and 15 m (Fig 2B).

## Discussion

In this study, we successfully developed a PCR-based method to detect plant DNA ingested by *A*. *lucorum*, and the high specificity and sensitivity to distinguish between cotton and mungbean indicates that this is a feasible approach for assessing the movement of mirid bugs at the landscape level.

Fast-evolving sequences of the chloroplast genome region make it a suitable DNA barcode to identify plants [18]. PCR assays using specific/general primers targeting chloroplast DNA have been directly applied to examine the food choices of herbivorous insects with chewing feeders [34–37] and sucking feeders on both plant cells [38] and on phloem sap [24]. Notably, when using chloroplast DNA for insect diet assessment, a trade-off occurs between amplification success and host plant resolution. Hereward and Walter suggested that the chloroplast *trn*L intron was not successfully amplified from target plant DNA in the green mirid bug *Creontiades dilutus* because of degradation by extra-oral digestion [38]. The region of P6 loop of the *trn*L intron is reportedly more robust for amplification from degraded DNA but exhibits lower host species resolution [32]. The mirid bug *A*. *lucorum* in this study resembles *C*. *dilutus* and *Lygus* spp. in feeding behavior; all of them perform extra-oral digestion and lacerate and macerate plant cells using stylet-probing movement and watery salivary discharge [44]. In addition, an objective of our study was to discriminate cotton from mungbean, the two defined host plants of *A*. *lucorum* [3–6], thereby both the amplification success and the resolution require fully consideration. We used two pairs of specific primers targeting the *trn*L-*trn*F region of chloroplast DNA to develop a DNA-based molecular diagnostic system. The sequence of the *trn*L-*trn*F region has been previously reported to show a high degree of variation across families and thus provides robust resolution when applied to systematic studies below the family level [45]. As expected, positive amplicons were observed, and the designed primers were confirmed to be specific for cotton and mungbean (S1 and S2 Figs).

Our feeding experiment revealed that the maximum-life for plant detection did not reach 100% at time = 0 h, which resembled previous reports [34, 37] and may be attributed to either the effect of extra-oral digestion in *A*. *lucorum* or the pretended ingestion of plant tissues. The detectability of cotton and mungbean DNA was significantly different, with cotton exhibiting a longer detectable time—its DS_50_ value was approximately two times higher than that of mungbean (Fig 1A and 1B). This result is consistent with findings for the *Agriotes* click beetle larvae [35], which suggest that plant identity can affect post-feeding DNA detection. Here, our results indicate that the mungbean digestion rate is higher than that of cotton. When feeding on the plants, mirid bugs typically discharge lytic salivary enzymes such as salivary polygalacturonases, proteases, amylases and glucose dehydrogenase that facilitate extra-oral digestion of host tissues [46]. Moreover, persuasive evidence indicates that mirid bugs like *Lygus hesperus* Knight prefer to feed on squares that have been preconditioned by salivary enzymes released during early stylet-probing activities [47]. In *A*. *lucorum*, previous experiments have shown that protease and amylase activities are significantly higher in mungbean-fed adults than in cotton-fed adults [48]. Additionally, the difference in digestion rates correlates to *A*. *lucorum* preference and performance for the two host plants, as both longevity and fecundity were improved in mirid bugs that fed on mungbean compared to those that fed on cotton [11]. Therefore, the different digestion rates for cotton and mungbean by *A*. *lucorum* adults might be partly ascribed to either distinct effects on catalytic digestive enzyme activity or a different relative fitness between these two host plants. However, these inferences require further investigation.

Molecular gut content analyses of ingested plant DNA can provide new insight into the landscape-level ecology of polyphagous insects. Recently, this technique has been demonstrated to be suitable for assessment on which host plants a polyphagous potato psyllid *Bactericera cockerelli* fed before the emergence of target host crops [24]. Our study revealed that the detectability of plant DNA decreased gradually with digestion time (Fig 1A and 1B), suggesting that the detection of both cotton and mungbean DNA was negatively correlated with the duration of digestion. Nevertheless, a weighted detection rate was used, which can correct for field data and compensate for different digestion times [49, 50]. According to this method, the weighted detection rates of field-collected bugs were comparable, and this finding could be important for confirming the presence of *A*. *lucorum* host plant species in open fields as well as for assessing their potential movement at the landscape level.

The mirid bug, *A*. *lucorum* resembled other mirid species like *C*. *dilutus* and *Lygus* spp. by exhibiting high mobility [51], and their observed occurrence on plants did not necessarily suggest regular feeding. The positive detection of ingested cotton and mungbean DNA in the guts of field-collected bugs indicated that *A*. *lucorum* adults in the field can feed on both cotton and mungbean, which was consistent with previous conclusions that both of them are *A*. *lucorum* host plants [3–6, 9]. In addition, the positive detection of cotton DNA in the guts of mirid bugs caught on mungbean plots suggested movement from cotton to the adjacent mungbean. This inference is more persuasive when the successively decreasing DNA detection rates for cotton from 5 to 15 m is compared with similar detection rates over distances for mungbean (Fig 2B). Likewise, based on molecular gut content analyses, Hereward and Walter proposed that the mirid bug *C*. *dilutus* often fed on host plant species other than the one from which it had been collected, indicating potential movements and multiple host usage of a mirid bug [38]. Our newly developed specific molecular diagnostic approach supported this inference; in addition to the specific detection of mungbean or cotton DNA, both mungbean and cotton DNA was detected in the gut of a single individual bug (Fig 2A). Interestingly, the ratio of bugs showing mixed feeding on both cotton and mungbean to bugs that were specifically cotton-fed was approximately 1:1, suggesting that half of the *A*. *lucorum* adults moved from cotton to the mungbean field fed on mungbean. This feeding habit was similar to another Hemipteran, *Nezara viridula* L, which moved from one plant species to another during feeding [52]. Velascol and Walter proposed that host-switching enhances survival and reproduction of *N*. *viridula* [53]. However, whether the ecological significance of *A*. *lucorum* movement resembles that in *N*. *viridula* or whether *A*. *lucorum* adult benefits from a combination diet under natural conditions still requires further investigation.

In conclusion, this study developed a molecular approach to identify remaining host plant DNA from the gut of a polyphagous mirid bug, *A*. *lucorum*. Our findings herein provide direct evidence supporting the movement of *A*. *lucorum* adults from cotton to mungbean fields and are a significant step toward exploiting the full potential of DNA-based molecular detection techniques to study the landscape-level ecology of *A*. *lucorum* under natural conditions.

## Materials and methods

### Insect rearing and plant collection

To perform the feeding experiment, a colony of *A*. *lucorum* has been established. Briefly, *A*. *lucorum* mirid bugs were obtained from a colony and reared on fresh green bean (*Phaseolus vulgaris*) pods in the laboratory at the Langfang Experimental Station at the Institute of Plant Protection of the Chinese Academy of Agricultural Sciences (IPP-CAAS). The colony was maintained at 25±1°C with 60±10% RH and a 16:8 h L:D photoperiod. To design specific primers and develop the molecular diagnostic system, the following host plants were selected: cotton, mungbean and 31 non-target plant species across 16 families (Table 2). These non-target plant species were all *A*. *lucorum* host plants and widely distributed across northern China. All plant specimens were collected at Langfang Experimental Station in the summer of 2014 and stored at -80°C until their use.

### Plant DNA extraction

Plant DNA was extracted from 1 cm diameter leaf discs using the DNeasy Plant Mini Kit (QIAGEN, Hilden, Germany) following the manufacturer’s protocol, and all the extracted DNA samples were quantified using a spectrophotometer (NanoDrop^™^ 1000, Thermo Fisher Scientific, USA) and stored at -20°C until use. To check for cross-sample contamination among extractions, two negative controls were included. General primers located in the *trn*L chloroplast DNA region—c B49317 primer (5’-CGAAATCGGTAGACGCTACG-3’, for the *trn*L (UAA) 5’ exon [30]) and *trn*L 110R primer (5’-GATTTGGCTCAGGATTGCCC-3’, for the *trn*L (UAA) intron [45]) function as a positive control to verify whether the plant DNA was well extracted, because they result in amplicons with similar sizes (approximate 120 bp) in different plant species.

### Primer design and specific assessment

The *trn*L-*trn*F region of the host cotton, mungbean and other non-target plants was aligned using the BioEdit sequence alignment editor 7.1.3.0 [54], and specific primers for cotton and mungbean were designed using Primer Premier 5 version 5.00 [55]. Plant sequences were obtained from GenBank, including sequences for *G*. *hirsutum* (HQ696725), *V*. *radiate* (JX233513) and the non-target plants. The specificity of the primers was tested by amplifying DNA from the leaves of those 31 non-target plant species by PCR (Table 2). The PCR products were analyzed on 2.0% agarose gels, purified using the Axygen Gel Extraction kit (Axygen) and cloned into the pGEM-T easy vector (Promega, Madison, WI, USA). Positive clones were selected by PCR and sequenced using an ABI3730XL automated sequencer (Applied Biosystems) with standard M13 primers.

### Feeding experiment

To test the sensitivity of the newly established molecular detection system, a feeding experiment was performed. Before the feeding trial, 3-day-old adult *A*. *lucorum* specimens were starved for 48 h to confirm no plant tissues remains within their guts (at 25±1°C). Tender cotton and mungbean leaves were separately placed in a Petri dish (10 cm diameter, 2.6 cm height). Thereafter, each adult bug was separately introduced into each Petri dish for 3 h at 25°C and then observed every 10 min (a mirid bug seen with its stylet inserted into the leaf at least three times was considered to have fed) [25]. After feeding, individuals were maintained at 25°C for 0, 2, 4, 8, 12, 16, 20 and 24 h (10 individuals per time point as a repetition, a total of three repetitions), and they were then frozen at -80°C until evaluation via PCR.

According to Wallinger et al. (2013) [35], the sensitivity of the PCR assays was assessed via serial dilution of template DNA (either the plant DNA template or the mirid bug DNA template extracted from samples in the feeding experiment). The results showed that no significant difference in primer efficiency within the same dilution ratio. No positive amplicons were observed at the lowest concentration tested by either the cotton or mungbean primers. A 4 μL extracted bug DNA solution (10 ng/μL) is able to provide positive amplicons for both cotton and mugbean primers.

### Insect DNA extraction

DNA from adult *A*. *lucorum* was extracted followed a CTAB-based protocol described by Wallinger et al. [35]. To avoid DNA amplification from plant material that may have adhered to the body surface of the mirid bugs, each adult mirid bug was cleaned following a modified method described in previous studies [35, 56, 57]. Briefly, each individual insect was placed in 1 mL of 1–1.5% sodium hypochlorite (Beijing Chemical Works, Beijing, China) for 5 s. Each individual was then rinsed twice with molecular-grade water. Our preliminary trial showed that this method successfully removed external plant DNA contamination and did not destroy the ingested plant DNA in the gut of *A*. *lucorum*. Two extraction-negative controls were included in each batch of 24 samples to check for cross-sample contamination.

### PCR assays

PCR amplifications were performed in 20 μL reaction mixtures containing 4 μL DNA solution (10 ng/μL), 2 μL 10× Taq buffer (TransGen Biotech, Beijing, China), 0.4 μL dNTP (2.5 mM), 0.2 μL Easy Taq (5 units/μL) (TransGen Biotech), 0.75 μL each primer (10 μM), and 11.9 μL autoclaved distilled water. The PCR reactions were performed in Veriti 96-Well Thermal Cyclers (Applied Biosystems, USA). The thermo cycling protocol began with an initial denaturing step of 95°C for 10 min, followed by 35 cycles of 95°C for 30 s, 56°C for 30 s and 72°C for 1 min, and a final extension of 72°C for 10 min. The PCR products (6 μL) were then separated using a 2% agarose gel in TAE buffer (40 mmol/L Tris-acetate, 2 mmol/L Na_2_EDTA H_2_O) and visualized with a UV trans-illuminator. Two positive (cotton or mungbean plant DNA) and two negative controls (PCR-grade water instead of extracted insect DNA) were included in each PCR assay to determine amplification success and DNA carry-over contamination, respectively.

### Field sampling

To assess the movement of *A*. *lucorum* adults from cotton to mungbean fields, field plots of cotton and mungbean were established at the Langfang Experimental Station of IPP-CAAS (39.53°N, 116.70°E) in Hebei Province, China in 2014. Cotton and mungbean plots pairs (20 m x 30 m, each) comprised a 20 m adjoining midline edge area (S3 Fig). Four replicates of these two host plant block (20 m x 60 m) were considered. The four blocks were arranged in a straight line from south to north, with 10 m intervals between adjacent blocks. A road and a wall were situated along the western side of the field blocks, a road was situated along the eastern side of the blocks, and roads and walls were situated at the northern and southern ends of the group of blocks. All the blocks received the same fertilizer and irrigation, and insecticide was not applied during the experimental period.

### Molecular detection of field-collected samples and distance effects

To confirm whether adult *A*. *lucorum* in mungbean plots migrated from the adjacent cotton plots and to evaluate the detection rate, we collected the adult *A*. *lucorum* in late July with sweep netting (38 cm diameter) in mungbean fields. The sampling range of each plot was 15 m away from the cotton-mungbean midline. Approximate sixty lively and undamaged adult bugs caught in each mungbean plot were subjected to molecular analysis using both mungbean- and cotton-specific primers. To assess the effect of distance, adult *A*. *lucorum* within each plot of mungbean were collected by sweep netting (38 cm diameter) along parallel lines 5, 10, and 15 m away from the cotton-mungbean midline. Each line was sampled by one hundred sweep nets (38 cm diameter), and the sampling depth was 20 m, i.e., the length of each designed plot. To avoid adult dispersal, sampling was performed simultaneously at each distance. Subsequently, approximately thirty lively and undamaged *A*. *lucorum* adults from each line were randomly selected for analysis. For molecular detection, each individual was transferred to a 1.5 mL microcentrifuge tube, transported back to the laboratory (within 1 h) on ice, and immediately frozen in liquid nitrogen. All the selected mirid bugs were stored at -80°C until their DNA was extracted. PCR assays were performed as previously described.

### Statistical analysis

The effect of digestion time (i.e., time post-feeding) on plant DNA detection success was tested for the species-specific plant primers using a logistic regression with a binomial distribution (PROC GENMOD). SAS 9.30 software was used to estimate the DS_50_ values [58] for the two plant species, which were compared using t-tests.

We used a previously described method [49, 50] to weight the plant detection rates and better interpret the extent of plant DNA in the gut contents under natural conditions. Briefly, the weighted detection rates were corrected based on the detection rates, the shorter DS_50_ of plant DNA was assigned an importance weighting value of 1.0, and the importance weighting value of the DNA of the other plant was obtained using this benchmark DS_50_ as the numerator and the DS_50_ of the other plant as the denominator. The corrected detection rate was calculated by multiplying the proportion of plant DNA in the field-collected *A*. *lucorum* adults by the importance weighting value. The weighted detection rates of the samples collected at different distances from the mungbean-cotton midline were compared via a one-way analysis of variance (one-way ANOVA).

## Supporting information

S1 Fig PCR amplification from all of the extracted plant DNA samples using general primers located in the *trn*L chloroplast DNA region. Lines 1–4, negative controls for DNA extraction; lines 5–6, negative controls for PCR amplification; lines 7–72, all of the plant species samples with two biological replicates. M is a 50 bp DNA ladder.
(TIF)

S2 Fig PCR amplifications from all of the extracted plant DNA samples using mungbean *V*. *radiata* (A) and cotton *G*. *hirsutum* (B) specific primers targeting the *trn*L-*trn*F region. Each sample includes two biological replicates. Lines 1 and 2, negative controls; lines 3 and 4, the targeted host plant species, mungbean or cotton; lines 5–66, 31 non-targeted host plants similar to the list in Table 2. M is a 50 bp DNA ladder.
(TIF)

S3 Fig Schematic diagram of the designed cotton and mungbean field plots. (TIF)
