# Supplementary figures and images for: The specific host plant DNA detection suggests a potential migration of Apolygus lucorum from cotton to mungbean fields
Source: PLoS One. 2017 Jun 6;12(6):e0177789. doi: 10.1371/journal.pone.0177789 (PMC5460846; doi:10.1371/journal.pone.0177789)

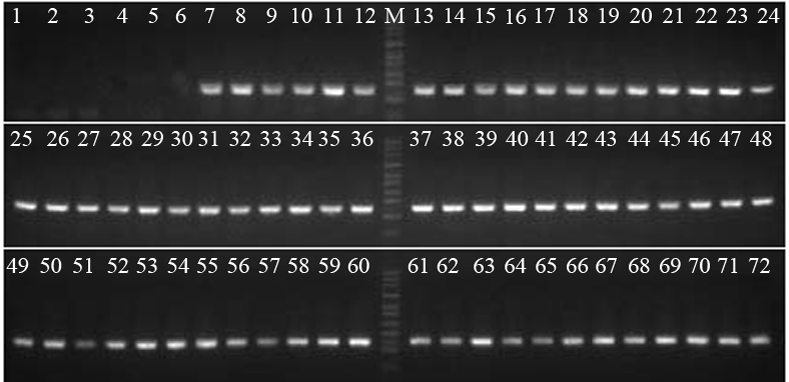

Supplement: S1 Fig — Lines 1–4, negative controls for DNA extraction; lines 5–6, negative controls for PCR amplification; lines 7–72, all of the plant species samples with two biological replicates. M is a 50 bp DNA ladder. (TIF) [file pone.0177789.s001.tif]

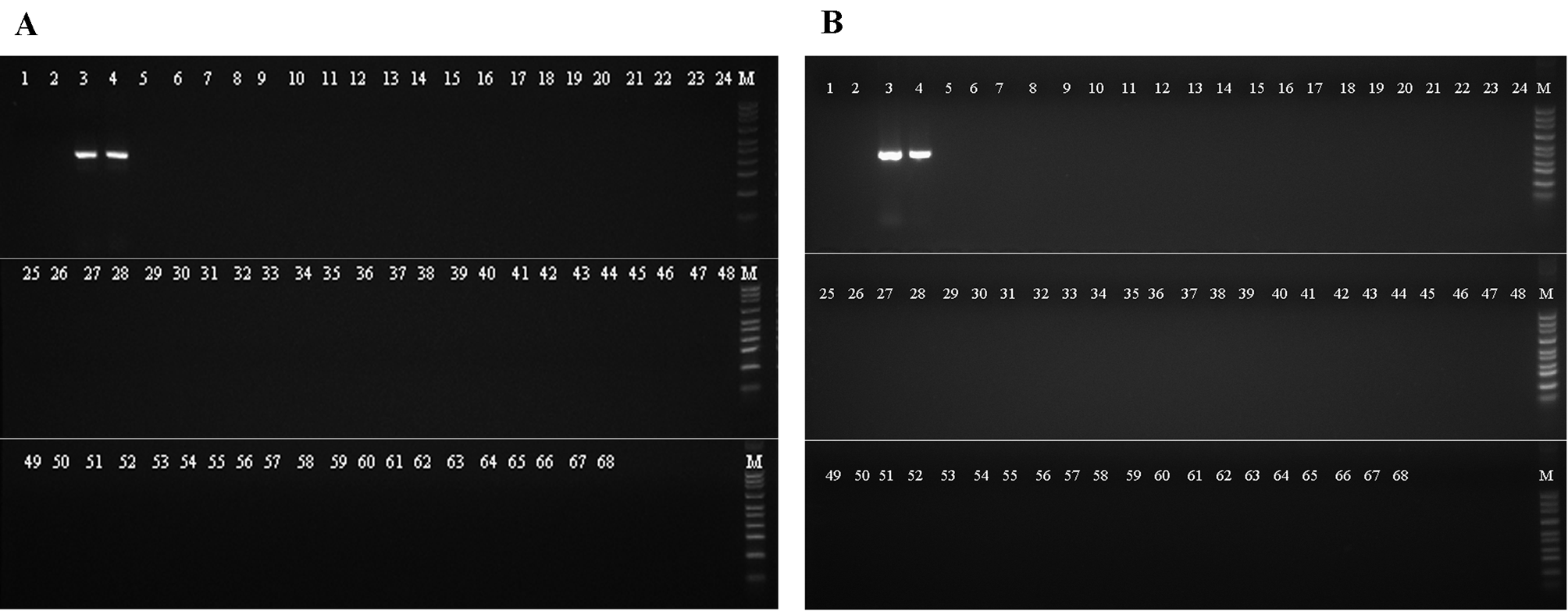

Supplement: S2 Fig — Each sample includes two biological replicates. Lines 1 and 2, negative controls; lines 3 and 4, the targeted host plant species, mungbean or cotton; lines 5–66, 31 non-targeted host plants similar to the list in Table 2. M is a 50 bp DNA ladder. (TIF) [file pone.0177789.s002.tif]

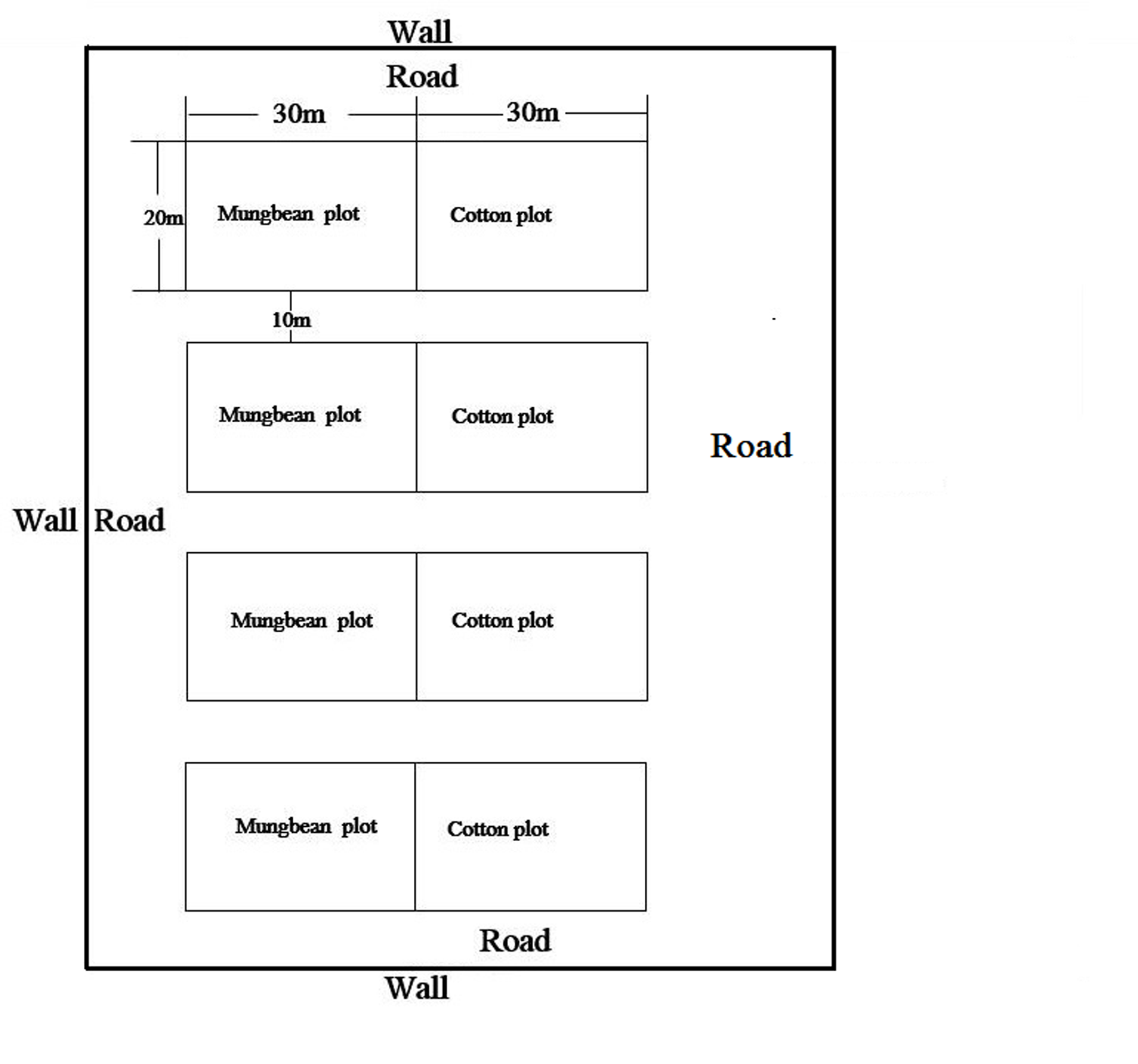

Supplement: S3 Fig — (TIF) [file pone.0177789.s003.tif]
